# DNA methylation array analysis identifies breast cancer associated *RPTOR*, *MGRN1* and *RAPSN* hypomethylation in peripheral blood DNA

**DOI:** 10.18632/oncotarget.11640

**Published:** 2016-08-26

**Authors:** Qiuqiong Tang, Tim Holland-Letz, Alla Slynko, Katarina Cuk, Frederik Marme, Sarah Schott, Jörg Heil, Bin Qu, Michael Golatta, Melanie Bewerunge-Hudler, Christian Sutter, Harald Surowy, Barbara Wappenschmidt, Rita Schmutzler, Markus Hoth, Peter Bugert, Claus R. Bartram, Christof Sohn, Andreas Schneeweiss, Rongxi Yang, Barbara Burwinkel

**Affiliations:** ^1^ Molecular Biology of Breast Cancer, Department of Gynecology and Obstetrics, University of Heidelberg, Heidelberg, Germany; ^2^ Division of Molecular Epidemiology (C080), German Cancer Research Center (DKFZ), Heidelberg, Germany; ^3^ Division of Biostatistics (C060), German Cancer Research Center (DKFZ), Heidelberg, Germany; ^4^ Department of Gynecology and Obstetrics, University Women's Clinic, Heidelberg, Germany; ^5^ Department of Biophysics, Center for Integrated Physiology and Molecular Medicine (CIPMM), Saarland University, Homburg (Saar), Germany; ^6^ Genomics and Proteomics Core Facility, German Cancer Research Center (DKFZ), Heidelberg, Germany; ^7^ Institute of Human Genetics, University of Heidelberg, Heidelberg, Germany; ^8^ Centre of Familial Breast and Ovarian Cancer, Department of Gynaecology and Obstetrics and Centre for Integrated Oncology (CIO), Center for Molecular Medicine Cologne (CMMC), University Hospital of Cologne, Cologne, Germany; ^9^ Institute of Transfusion Medicine and Immunology, Medical Faculty Mannheim, University of Heidelberg, German Red Cross Blood Service Baden, Württemberg, Hessen, Mannheim, Germany; ^10^ National Centre for Tumor Diseases, Heidelberg, Germany

**Keywords:** breast cancer, DNA methylation, MGRN1, RAPSN, RPTOR

## Abstract

DNA methylation changes in peripheral blood DNA have been shown to be associated with solid tumors. We sought to identify methylation alterations in whole blood DNA that are associated with breast cancer (BC). Epigenome-wide DNA methylation profiling on blood DNA from BC cases and healthy controls was performed by applying Infinium HumanMethylation450K BeadChips. Promising CpG sites were selected and validated in three independent larger sample cohorts via MassARRAY EpiTyper assays. CpG sites located in three genes (cg06418238 in *RPTOR*, cg00736299 in *MGRN1* and cg27466532 in *RAPSN*), which showed significant hypomethylation in BC patients compared to healthy controls in the discovery cohort (*p* < 1.00 × 10^−6^) were selected and successfully validated in three independent cohorts (validation I, n =211; validation II, n=378; validation III, n=520). The observed methylation differences are likely not cell-type specific, as the differences were only seen in whole blood, but not in specific sub cell-types of leucocytes. Moreover, we observed in quartile analysis that women in the lower methylation quartiles of these three loci had higher ORs than women in the higher quartiles. The combined AUC of three loci was 0.79 (95%CI 0.73-0.85) in validation cohort I, and was 0.60 (95%CI 0.54-0.66) and 0.62 (95%CI 0.57-0.67) in validation cohort II and III, respectively. Our study suggests that hypomethylation of CpG sites in *RPTOR*, *MGRN1* and *RAPSN* in blood is associated with BC and might serve as blood-based marker supplements for BC if these could be verified in prospective studies.

## INTRODUCTION

Breast cancer (BC) is the second most common cancer in the world and the most frequent cancer among women. It is the leading cause of cancer mortality for women [1]. In 2016, it is estimated that there will be 246,660 new cases of female BC and an estimated 40,450 people will die of this disease [2]. Although therapeutic advances have improved the survival rate of this disease, many BC patients still suffer from greatly reduced quality of life or develop metastasis due to late diagnosis [3].

Based on known risk factors including age and reproductive, medical and family history, the Gail model is developed. It is a popular BC risk prediction method currently available for populations, but the predictive accuracy of this model for BC in individuals is only about 58.0% to 59.0% [4, 5]. Rare inherited mutations of the BC susceptibility genes, such as *BRCA1/2*, *P53*, *PTEN*, *CHEK2* and *ATM*, are strongly associated with familial breast cancer, but together only account for 1.5-3% of all BCs [6]. Although recent genome-wide association studies have identified multiple variants with low-penetrance risk to BC, a panel of 10 such SNPs has a predictive accuracy of only 59.7% [5]. Therefore, the known lifestyle, environmental and genetic risk factors have limited use in predicting a woman's risk of BC.

Aberrant DNA methylation is a critical mechanism in carcinogenesis [7, 8]. Dysregulation of tumor DNA methylation, such as hypermethylation of CpG islands at the promoters of tumor suppressor genes and global hypomethylation have been observed in almost every cancer type [9]. Although DNA methylation profiles are often tissue and cell-type specific, recent data indicate that epigenetic changes in blood cell DNA are potential markers for solid tumors [10–15]. Previous reports of associations between blood DNA methylation and cancer include studies of global DNA methylation levels in repetitive regions across the genome (e.g. LINE1, Alu) and 5-mC content in genomic DNA [16, 17]; studies of gene-specific DNA methylation levels in candidate genes [18, 19], and genome-wide DNA methylation microarray studies [20–22]. A review and meta-analysis concluded that DNA methylation in peripheral blood cells has a great potential as a supplement for cancer biomarkers [23, 24].

Candidate gene studies have reported associations between BC risk and methylation of *ATM* and *BRCA1* genes in peripheral blood [10, 18, 19, 22, 25]. Most recently, two large genome-wide studies used blood samples collected before diagnosis have reported associations between BC risk and epigenome-wide hypomethylation of blood DNA [20, 21]. Our previous study has also suggested an association between decreased *HYAL2* methylation in the peripheral blood and increased probability of having BC [26]. In the present study, we first created epigenome-wide DNA methylation profiles of peripheral blood from a case-control study, aiming to identify the strongest methylation changes in blood DNA that are associated with BC. The identified CpG sites were further validated in three independent sample cohorts (Table 1) by MassARRAY EpiTyper assays.

**Table 1:** Sample cohorts used in this work

Study Phase	Sample Description	Number	Age (mean ± SD, y)	Assays
Discovery/Replication	Sporadic BC cases	48	47.7 ± 7.2	Human450K methylation array/MassARRAY
	Healthy controls	48	46.7 ± 7.5	
Validation I	Sporadic BC cases	109	46.6 ± 7.4^a^	MassARRAY
	Healthy controls	102	42.6 ± 16.5^a^	
Validation II	Sporadic BC cases	189	59.6 ± 11.7	MassARRAY
	Healthy controls	189	59.1 ± 9.4	
Validation III	Familial BC cases	270	44.3 ± 9.3	MassARRAY
	Healthy controls	250	44.8 ± 9.6	

a There is significant difference of age between cases and controls (t-test, *p* = 0.024)

## RESULTS

### Discovery of BC-associated DNA methylation signatures by Illumina 450K DNA methylation array

We first performed a genome-wide DNA methylation screening on blood-based DNA in the discovery cohort with 48 sporadic BC cases and 48 controls using the Illumina 450K DNA methylation array. We observed the trend towards marginally reduced global DNA methylation levels in BC patients compared to controls (mean β of cases: 52.33%, mean β of controls: 52.41%, *p* = 0.089), which is in agreement with previous studies [20, 21]. To avoid spurious associations, we excluded from downstream analysis any loci with SNPs overlapping the Illumina probe sequence according to the HumanMethylation450K annotation files, leaving 392,370 probes [27]. Supplementary Figure S1 shows the raw *p* values for all 392,370 CpG sites versus mean methylation differences between groups. Since a great number (n = 136) of CpG sites retained a significant methylation difference after correction for multiple testing, a more detailed strategy was applied to select the CpG sites for validation: 1) raw *p* value ≤ 1.40E-06, corresponding to a false discovery ratio of 0.005; 2) mean methylation difference between cases and controls (Δβ) > 4 %; and 3) CpG site is not an intergenic site. This resulted in 20 CpG sites across 17 genes. Of these, seven CpG sites in seven genes from the top, which the surrounding DNA sequence fulfilled the requirements of assay design for the MassARRAY Epityper assay were considered (Table 2).

**Table 2:** Methylation levels of respective CpG sites of seven genes in replication and pre-validation round

	CpG loci	Gene	Replication (48 BC cases vs 48 healthy controls)		Initial validation^a^ (47 BC cases vs 47 healthy controls)	
			Methylation difference (case –control)	*p* value^b^	Methylation difference (case –control)	*p* value^b^
1	cg06418238	*RPTOR*	−0.09	**1.21E-04**	−0.05	**0.013**
2	cg00736299	*MGRN1*	−0.05	**0.011**	−0.09	**0.002**
3	cg27466532	*RAPSN*	−0.07	**0.020**	−0.09	**0.011**
4	cg06526620	*FUT4*	−0.04	**0.015**	−0.02	0.296
5	cg21932542	*RADIL*	−0.10	**1.98E-04**	−0.03	0.357
6	cg22941668^c^	*MIR145*	0.01	0.586	n.d.	n.d.
7	cg22233512^c^	*MSI2*	−0.05	0.203	n.d	n.d.

a Samples are from validation cohort I

b *p* values are calculated by logistic regression, adjusted by age. Significant *p* values are in bold

c These two CpG sites failed in the replication round and were not considered for initial validation. n.d., not done

To test for the influence of cellular heterogeneity, the method developed by Houseman et al. [28] and the Reinius reference dataset [29] were implemented to first estimate the proportions of six different sub cell type of leucocytes for each sample and then adjust for those in the beta regression. We observed small but significant differences in the proportions of CD4^+^ T cells (0.14 vs 0.17, *p* = 0.005) and granulocytes (0.66 vs 0.61, *p* = 0.0001) between BC cases and controls, but no differences in the proportion of CD8^+^ T, NK, B cells or monocytes (Supplementary Table S1). After adjustment for the cell type proportions, these seven CpG sites still showed significantly decreased methylation levels in BC cases compared to controls (Supplementary Table S2) and were thus selected for validations.

### Validation of BC associated altered methylation in *RPTOR*, *MGRN1* and *RAPSN* CpG loci in three validation cohorts

For the seven candidate CpG loci we first replicated the methylation results with MassARRAY EpiTyper assays on the same samples used in the 450K BeadChips (Table 2). The observed methylation differences between BC cases and controls could be verified in five out of seven genes (Table 2). These CpG sites were chosen for further independent validations. All samples used in validations had not been used in the 450K BeadChips analysis.

**Figure 1:**
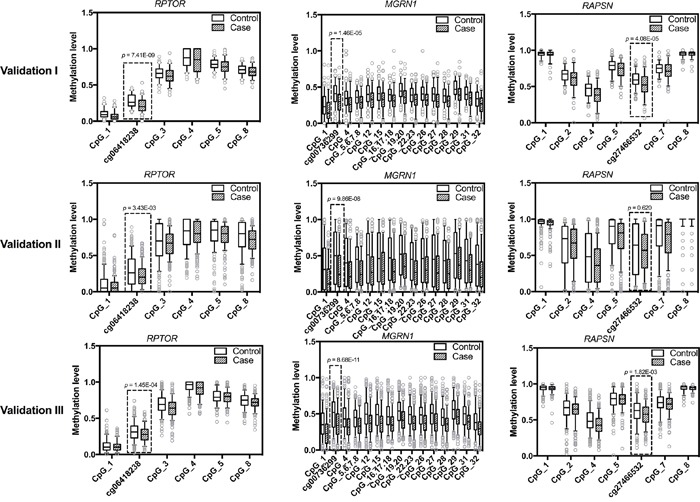
Methylation levels of *RPTOR* (cg06418238), *MGRN1* (cg00736299), *RAPSN* (cg27466532) and adjacent CpG sites in study population The box plots show the distribution of methylation levels of the three loci identified by 450K array (framed in boxed) and adjacent CpG sites in three validation rounds by MassARRAY, respectively. Validation cohort I contains 109 sporadic BC cases and 102 healthy female controls; Validation cohort II contains 189 sporadic BC cases and 189 controls from an independent study cohort; Validation cohort III contains 270 familial BC cases and 250 controls. The *p* values were calculated by logistic regression adjusted for age and different experimental batches. The circles indicate outliers.

Validation cohort I consisted of 109 sporadic BC cases and 102 healthy controls (Table 1). In an initial analysis of 47 BC cases and 47 controls, only cg06418238 in *RPTOR*, cg00736299 in *MGRN1* and cg27466532 in *RASPN* could be verified (Table 2). Thus, these three CpG loci were chosen for analysis in the remaining of samples of validation cohort I as well as for validation in cohorts II and III. In validation cohort I, the three CpG sites showed significantly lower median methylation in BC cases than in controls (Figure 1). The same could be observed with most of the adjacent CpG sites additionally present in the amplicons (Figure 1). Quartile analysis revealed that ORs for women in the lowest methylation quartile for *RPTOR* (cg06418238), *MGRN1* (cg00736299) and *RAPSN* (cg27466532) loci were 5.29 (95% CI 2.36-11.86), 6.22 (95% CI 2.69-14.36) and 2.97 (95% CI 1.34-6.56), compared with women in the highest quartile, respectively (Supplementary Table S3).

Validation cohort II included 189 sporadic BC cases and 189 controls from an independent study cohort (Table 1). Due to the shortage of DNA materials, only a minimal amount of DNA (about 5 ng) was used for PCR and further analysis, which might be the reason why the MassArray results featured a wider range in the validation cohort II than the validation cohort I (Figure 1). Despite increased inter-quartile ranges (IQR), the overall pattern of group differences with pronounced hypomethylation in cases was unambiguously preserved (Figure 1). In concordance to validation cohort I, quartile analysis also showed increasing frequencies of BC patients with lower methylation intensities (Supplementary Table S3). The ORs for women in the lowest methylation level quartile of *RPTOR* (cg06418238) and *MGRN1* (cg00736299) loci were 1.95 (95% CI 1.09-3.48) and 3.31 (95% CI 1.79-6.14), respectively, compared to women in the highest methylation level quartile. For the *RAPSN* (cg27466532) locus there was no such association. However this is likely due to increased IQR ranges in this cohort since the second and third quartiles showed significantly increased ORs for BC compared to the lowest quartile (Supplementary Table S3).

We further validated the candidates in 270 familial BC patients and 250 healthy controls (Table 1, validation cohort III), to check if hypomethylation of the respective CpG sites of these genes was also associated with familial breast cancer. In line with the observations in the previous cohorts, reduced methylation levels in BC cases were observed in the targeted CpG loci and surrounding CpG sites (Figure 1), with the fraction of BC cases again increasing with lower methylation intensities (Supplementary Table S3). For *RPTOR* (cg06418238), *MGRN1* (cg00736299) and *RAPSN* (cg27466532) loci, ORs for women in the lowest methylation levels quartile were 2.52 (95% CI 1.54-4.11), 4.61 (95% CI 2.73-7.76) and 2.05 (95% CI 1.26-3.34), respectively, compared to women in the highest quartile.

### Combination analysis of associations between methylation levels of *RPTOR* (cg06418238), *MGRN1* (cg00736299), *RAPSN* (cg27466532) and breast cancer

Combining MassARRAY EpiTyper methylation data from all validation cohorts revealed that the overall differences in median methylation levels between BC cases and healthy controls were 4% for cg06418238 (*p* = 2.52E-08), 17% for cg00736299 (*p* = 2.32E-19) and 6% for cg27466532 (*p* = 1.01E-04) (Table 3). The ORs for *RPTOR* (cg06418238), *MGRN1* (cg00736299) and *RASPN* (cg27466532) loci were 2.81(95% CI 1.97-4.01), 5.14 (95% CI 3.48-7.60) and 2.04 (95% CI 1.45-2.88) for women in the lowest quartiles compared to women in the highest quartiles (Table 3).

**Table 3:** Combination analysis of associations between methylation levels of *RPTOR* (cg06418238), *MGRN1* (cg00736299), *RAPSN* (cg27466532) and breast cancer

Gene		Combined analysis			
	Methylation^a^	Control N^b^	Case N^b^	OR (95% CI)	*p* - value^c^
*RPTOR*	median (IQR)	0.28 (0.19-0.40)	0.24 (0.15-0.33)		**2.52E-08**
(cg06418238)	Q1 (<=0.19)	142	207	2.81 (1.97 -4.01)	
	Q2 (0.19-0.28)	113	131	2.26 (1.54 -3.32)	
	Q3 (>0.28-0.40)	147	158	2.09 (1.45 -3.02)	
	Q4 (>=0.40)	139	72	1.00 (reference)	
	*p* for trend				**<0.001**
*MGRN1*	median (IQR)	0.48 (0.28-0.67)	0.31 (0.16-0.48)		**2.32E-19**
(cg00736299)	Q1 (<=0.28)	135	248	5.14 (3.48 -7.60)	
	Q2 (0.28-0.48)	134	174	3.71 (2.48 -5.54)	
	Q3 (>0.48-0.67)	136	94	1.97 (1.29 -3.00)	
	Q4 (>=0.67)	135	49	1.00 (reference)	
	*p* for trend				**<0.001**
*RAPSN*	median(IQR)	0.62 (0.47-0.75)	0.56 (0.44-0.69)		**1.01E-04**
(cg27466532)	Q1 (<=0.47)	141	193	2.04 (1.45 -2.88)	
	Q2 (0.47-0.62)	129	158	1.86 (1.30 -2.66)	
	Q3 (>0.62-0.75)	136	127	1.42 (0.98 -2.04)	
	Q4 (>=0.75)	135	90	1.00 (reference)	
	*p* for trend				**<0.001**

Abbreviation: IQR, interquartile range

a Methylation quartiles are based on methylation distributions on all control samples

b Differences in numbers of cases and controls with total numbers of the study are due to missing data on methylation markers

c Mann-Whitney U test for median methylation differences between groups and logistic regression for the trend test, bold signifies *p* < 0.05

### Association of altered methylation in *RPTOR, MGRN1* and *RAPSN* and clinical characteristics of BC

The methylation levels of the three representative CpG sites did not show any association with age, menopause status, ER/PR status, HER2 status, grading, tumor size, lymph node status or tumor stage in the sporadic BC patients with available clinical data (Supplementary Table S4).

### Methylation of CpG sites in *RPTOR*, *MGRN1* and *RAPSN* in different types of leucocytes

DNA samples used in the present study are derived from a complex mixture of functionally and developmentally component cell types with unique DNA methylation signatures. We studied the contribution of methylation differences in different cell populations to the observed consistent methylation differences by analyzing methylation levels in sorted cell populations. B cells, T cells and the B/T –lymphocytes-depleted leucocytes were separated from blood of seven sporadic BC cases and 13 healthy controls. Among all the investigated CpG sites, three CpG loci identified from the 450K array, as well as CpG_22.23, CpG_27 in *MGRN1* and CpG_5 in *RAPSN* showed significant methylation differences between BC cases and controls in whole blood (Supplementary Table S5). For the B cell fraction, T cell fraction and B/T-cells depleted leucocytes, no methylation differences were observed in all the investigated CpG sites. This suggests that the decreased methylation differences we observed in BC cases are not likely due to methylation changes in one specific cell type.

### Receiver operating characteristic analysis

**Figure 2:**
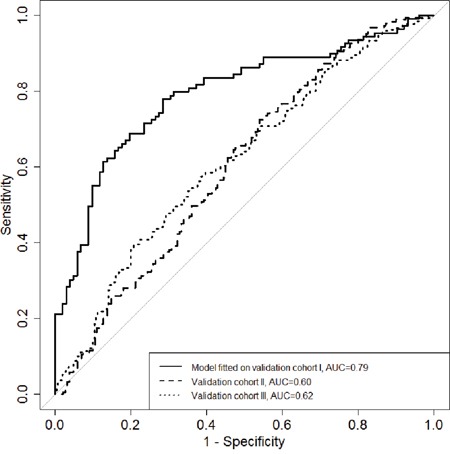
The diagnostic potential of the combined maker panel (*RPTOR*, *MGRN1* and *RAPSN*) for differentiating breast cancer cases from healthy controls ROC curves for logistic regression models based on the combination of *RPTOR*, *MGRN1* and *RAPSN* in three validation rounds. Backwards conditional variables selection method was used in the logistic regression.

To estimate the potential power of these three genes with regard to differentiate the BC cases from the controls, ROC curve analysis was performed by logistic regression using the backwards conditional variable selection method and adjusting for age and experimental batches. The model was built on the data set of validation cohort I, revealing an internal AUC of 0.79 (95%CI 0.73-0.85) and validated externally in both validation cohorts II and III with AUCs of 0.60 (95%CI 0.54-0.66) and 0.62 (95%CI 0.57-0.67), respectively (Figure 2).

## DISCUSSION

In the present study, we observed overall reduced methylation levels in blood DNA from BC patients compared to healthy controls. Three CpG sites (cg06418238 in *RPTOR*, cg00736299 in *MGRN1* and cg27466532 in *RAPSN*) were successfully validated in three independent study cohorts. Results of all three rounds of validation confirmed a significant decrease of methylation of these three loci in the peripheral blood DNA of both sporadic and familial BC cases compared to healthy controls. These findings applied not only for the CpG sites in *RPTOR*, *MGRN1* and *RAPSN* discovered by Illumina 450K profiling, but also for the adjacent CpG sites that were included in the regions analyzed by MassARRAY EpiTyper assay. The failure of validation for the other CpG sites identified from the Humanmethylation 450K data also suggested that an independent validation is necessary for further application of array-based data.

Blood-based epigenetic variation may be observed due to shifts in leukocyte populations [30]. To check this, we estimated six cell type proportions for the samples used in the 450K BeadChips according to the Houseman [28] method and data published by Reinius [29] and adjusted 450K array data for this. We found that the CpG loci still fulfilled the criteria for stratification. To exclude that only changes in the epigenetic profile of one specific subpopulation are associated with BC in the validation cohorts, we determined the methylation levels of all investigated loci in several subpopulations of leucocytes. Despite the small sample size, methylation differences were observed in whole blood, but not in any specific subpopulation of leucocytes.

The regulatory-associated protein of mTOR, complex 1 (*RPTOR*), also known as raptor, is an important scaffolding protein that recruits mTOR substrated to rapamycin-sensitive mTOR complex 1 (mTORC1) [31]. Raptor is required for the suppressive function of regulatory T cells (T_reg_) *in vivo* [32]. *MGRN*1 (mahogunin ring finger 1, E3 ubiquitin protein ligase) is a C3HC4 RING-containing protein with E3 ubiquitin ligase activity *in vitro*. Loss of function of this gene leads to late-onset spongiform neurodegeneration [33]. The receptor-associated protein of the synapse (*RAPSN*) gene encodes proteins that are receptors associated proteins of the synapse. Diseases associated with *RAPSN* include fetal akinesia deformation sequence and postsynaptic congenital myasthenic syndromes [34]. Further, *RAPSN* has been shown to play an important role in lysosome positioning, exocytosis and plasma membrane integrity [35]. In our study, we observed significantly lower methylation of respective CpG sites of the three genes in blood DNA of BC patients than that of cancer-free controls. However, the functions of these genes in hematopoietic system are still unknown. Future studies for the mechanisms of these genes in blood cells, or even immune cells may provide hints for the initiation and progression of cancer.

The FDA approved blood-based biomarkers for BC, such as CA15-3 and CA 27-29, are recommended for the monitoring of disease relapse and treatment efficacy, rather than diagnosis [36, 37]. For screening of hereditary BC, which constitutes only about 5-10% of total BC cases [38], there are *BRCA1/2* mutation analysis [39]. For sporadic BC, some new molecular tests namely particular sets of SNPs [40, 41] were available as supplements for mammography. However, the present screening methods are criticized by both low sensitivity [41, 42] and overdiagnosis disadvantages [43, 44]. Methylation changes of CpG sites in *RPTOR*, *MGRN1* and *RAPSN* were not associated with age or other clinical characteristics in affected individuals (Supplementary Table S4) and thus have similar power across different subtypes and stages of BC. With these characteristics, these markers might be useful in the aspect of BC risk stratification, if they could be successfully verified in prospective studies.

The main strength of this study is the inclusion of three independent study cohorts, the matched design, use of blood samples collected before any treatment, the standardized and short blood sample processing time and the plating of case-control pairs on the same chip to reduce the impact of technical variability of methylation level on the estimates of association. A further strength of the present study is the use of the HumanMethylation450K BeadChips to explore genome-wide DNA methylation profiles combined with the use of quantitative MassARRAY EpiTyper assay to very specific sites of interest. However, our study has limitations that are related to retrospective study cohorts and the relatively small sample set used in our 450K Beadchip discovery cohort. To address the latter issue, we included a large number of subjects in three independent validation cohorts. A large portion of our investigated breast cancer patients were at early stage (stage 0-II). So we can (at least) assume that lower methylation of the investigated CpG sites has already occurred in early period of breast cancer. This phenomenon could have happened before the onset of the disease and been preserved, or just happened at the early stage of the disease. We could not distinguish these two possibilities from our case –control study design. In addition, we didn't observe significant correlation between methylation level of the investigated CpG sites and breast cancer stage in our study (Supplementary Table S4). Thus we refrained from arguing that the lower methylation of the investigated CpG sites occurred in early period of breast cancer. In addition, as the combined AUCs of the three loci identified by the present study are not extremely high, thus we cannot judge that these three loci have better performance for BC risk evaluation than the other reported makers, such as *HYAL2* and *ATM*. It is also worth to point out here that receiver operating characteristic AUC fitted/modeled on Validation Cohort I samples was 0.79, but when this model was applied to calculate AUCs for Cohorts II and III, the AUCs were of 0.60 and 0.62, respectively. This suggests that independent validations of data/models are extremely necessary. Further, prospective studies are needed. It has been reported in two prospective studies that altered DNA methylation in blood DNA of BC patients could be detected years before the onset of the disease [20, 21]. Thus, in general blood DNA methylation markers have great potential and advantages to subserve BC early detection and risk evaluation. However we are still at an early stage. Results need to be verified in large prospective and population based studies to identify the most informative and most stable combination of different BC associated methylation sites and to figure out if the combination with other BC associated molecular blood based markers would improve such marker set.

In conclusion, our study documented for the first time that lower methylation of CpG sites in *RPTOR*, *MGRN1* and *RAPSN* in peripheral blood DNA is associated with sporadic as well as familiar BC. These might be part of a non-invasive blood-based molecular marker set for the evaluation of BC risk or early detection.

## MATERIALS AND METHODS

### Study populations

This study was approved by the Ethics Committee of the Medical Faculty in Heidelberg. The study population has been previously described [26, 45]. Briefly, all peripheral blood samples from BC cases and healthy controls were obtained from centers in Southwest Germany. All cases and controls were Caucasians and gave written informed consent. All peripheral blood samples from BC cases were collected at the time point of diagnosis before they received any therapeutic treatments. Clinical characteristics of BC patients were defined according to the American Joint Committee on Cancer staging manual [46]. The study cohorts used in this work are listed in Table 1 and briefly described below.

#### Patients and healthy controls for the discovery cohort and validation cohort I

Blood samples from surgically confirmed BC patients were collected between 2010 and 2012 at the time-point of diagnosis at the University Hospital of Heidelberg. Control blood samples were collected between 2010 and 2012 from healthy women. The blood samples from the patients and controls in the validation cohort I were processed in parallel with the same protocol.

#### Patients for validation cohort II

Peripheral blood samples were consecutively collected at the University Hospital of Heidelberg. A total of 189 sporadic BC samples collected in the years of 2009 and 2010 were randomly selected for our study. Characteristics of sporadic BC cases for the discovery cohort and validation cohorts I and II are described in Table 4.

**Table 4:** Characteristics of sporadic BC patients

Characteristics	450K Discovery /Replication	Validation I	Validation II
	n	n	n
Menopause status			
premenopausal	26	66	40
perimenopausal	7	9	16
postmenopausal	15	28	124
unknown	0	6	9
ER status^a^			
negative	7	17	23
positive	41	86	162
unknown	0	6	4
PR status^a^			
negative	10	25	38
positive	38	78	148
unknown	0	6	3
HER2_NEU status^b^			
negative	41	75	166
positive	7	28	19
unknown	0	6	4
Histological tumor grading			
I	8	12	35
II	35	59	114
III	5	31	38
unknown	0	7	2
Tumor size			
IS(*in situ*) and pT1	26	53	119
pT2	18	41	57
pT3 and pT4	4	8	12
unknown	0	7	1
Lymph nodes			
N0	35	69	132
N1-N3	13	31	53
unknown	0	9	4
Stage			
0 and I	23	39	101
II	20	50	61
III and IV	5	14	26
unknown	0	6	1

a Immunoreactive score (IRS) 0–2 was defined as ER/PR negative and 3–12 as ER/PR positive

b HER-2 IHC-score 0–1 was defined as HER2 negative and 3 as definitely positive. An IHC-score equal to 2 was further analyzed by FISH/CISH and deemed positive if HER2 was amplified

#### Patients for validation cohort III

Peripheral blood samples from *BRCA1/2* mutation negative index familial BC patients were collected by the centers of the German Consortium for Hereditary Breast and Ovarian Cancer (GC-HBOC) in Heidelberg and Cologne during the years 1997–2007. All the familial BC cases were recruited according to the criteria of family history described previously [47]. A total of 270 familial BC samples were randomly selected for our study. Characteristics of familial BC cases are not available

#### Controls for validation cohort II and III

Peripheral blood samples were consecutively collected from blood donors by the German Red Cross Blood Service of Baden-Württemberg-Hessen (Mannheim, Germany). Blood donors agreed on the use of their blood samples for research purposes. All control individuals were healthy at the time of blood donation during the years 2004–2010. Additionally, none of the control individuals had a reported family history of BC. DNA of a total of 439 healthy and unrelated females was randomly selected via the DNA bank of the Red Cross Blood Service as controls for validation cohorts II and III. There was no overlap between control samples used in validation II and III cohorts.

### DNA Methylation assessment

### Infinium HumanMethylation450K BeadChip

The Infinium HumanMethylation450K BeadChip [48] (Illumina, San Diego, CA, USA) analysis was conducted according to the manufacturer's instructions at the Genomics and Proteomics Core Facility of the German Cancer Research Center (DKFZ) in Heidelberg, Germany. In brief, DNA samples extracted from whole blood were bisulfite converted, purified and applied to the BeadChips. Image processing and intensity data extraction were performed according to Illumina's instructions. Methylation at each CpG site is described as β value [β = intensity of the methylated allele (M) / (intensity of the unmethylated allele (U) + intensity of the methylated allele (M) + 100)] [49]. It is expressed as a continuous variable that ranges from 0 (no methylation) to 1 (full methylation).

### MassARRAY EpiTyper assay

In validation rounds, the Sequenom MassARRAY EpiTyper assay was applied as described previously [26, 50]. DNA methylation levels at each CpG locus were determined by comparing the signal intensities of methylated and non-methylated templates. Sequences of the investigated regions are shown in Supplementary Table S6. Primers for the PCR amplifications are available upon request.

### Blood cell fractionation from leucocytes

Leucocytes were freshly isolated from peripheral blood from seven sporadic BC patients and 13 female healthy controls using red blood cell lysis buffer within 2 hours of blood collection [26]. First, B cells were positively isolated using a Dynal® CD19 positive isolation kit (Invitrogen, USA) from fresh leucocytes. Subsequently these B cell-depleted leucocytes were applied for T-cell purification with a Dynal® CD3 positive isolation kit (Invitrogen, USA). The leftover cells were collected as “B/T-cells depleted leucocytes”. The cell pellets were snap frozen in liquid nitrogen after purification and kept at −80°C until use. DNA was isolated from the different blood cell types using AllPrep DNA/RNA/Protein Mini Kit from Qiagen (Germany).

### Statistical analysis

The Illumina 450K BeadChips data were processed by the Illumina BeadStudio software with default settings. Probes with detection *p* –value > 0.01 were removed and samples were quantile-normalized. Association of probes with case – control status was evaluated by beta-regression models with a logistic link and associated Wald tests using the R package *betareg*. Likelihood Ratio tests were applied to compare the case-control model with nested model for chip differences. To control the false discovery rate, multiple testing was performed using the *Benjamini-Hochberg* method. The cell-type proportions were adjusted for the Illumina 450K data by fitting data linear mixed effects models [28]. All statistical analyses for Methylation450K data were conducted in R 3.2.3.

For MassARRAY Epityper data, age-adjusted *p* values were computed from Wald tests in logistic regression models including age and experimental batches as covariates. Corresponding area under the curve (AUC) was calculated. The conduct of the study and the report and analysis of the data were followed the REMARK criteria [51, 52]. All the statistical analyses of MassARRAY data were conducted in SPSS Statistics 22.0.

## SUPPLEMENTARY FIGURE AND TABLES




