# Supplementary material for: DNA methylation array analysis identifies breast cancer associated RPTOR, MGRN1 and RAPSN hypomethylation in peripheral blood DNA
Source: Oncotarget. 2016 Aug 26;7(39):64191–202. doi: 10.18632/oncotarget.11640 (PMC5325435; doi:10.18632/oncotarget.11640)
Supplement: Supplementary file 1 [file oncotarget-07-64191-s001.pdf]

DNA methylation array analysis identifies breast cancer associated *RPTOR*, *MGRN1* and *RAPSN* hypomethylation in peripheral blood DNA

SUPPLEMENTARY FIGURE AND TABLES

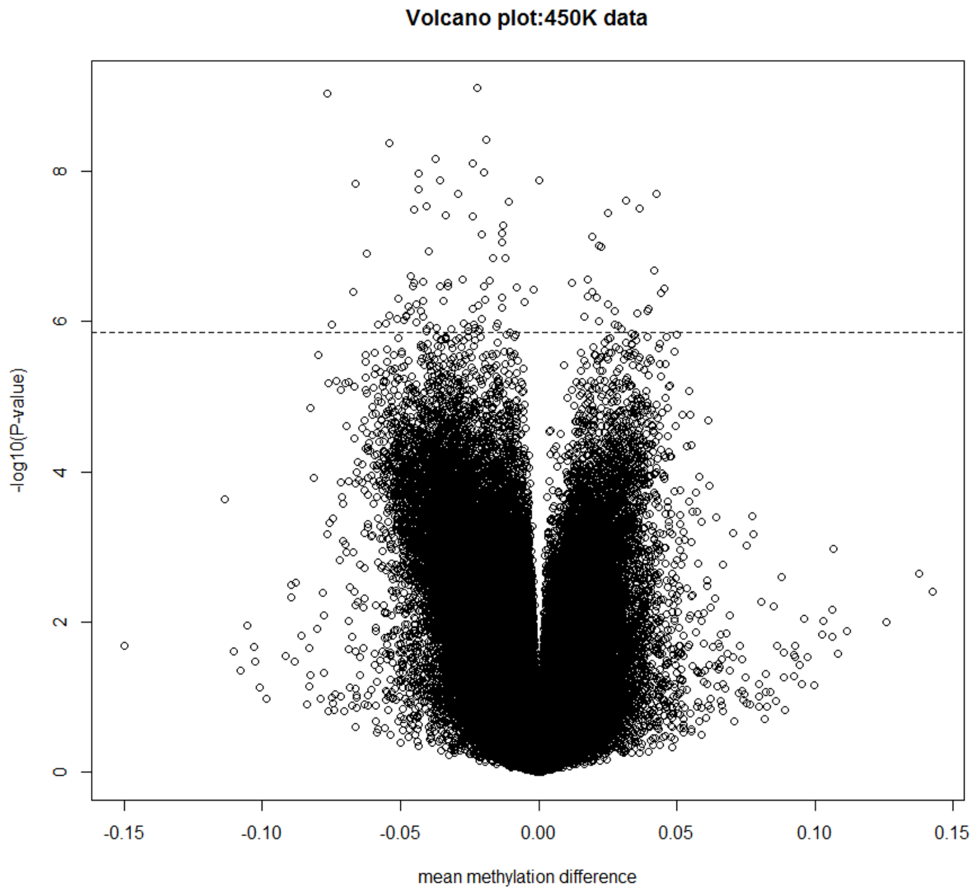

Supplementary Figure S1: Volcano plot for the Infinium HumanMethlyation 450K array results. The dotted line indicates the threshold of *p* =1.4E-06 (false discovery rate (FDR) =0.005).

Supplementary Table S1: Cell proportion estimates (mean ± SD) in BC cases and controls

Celltype[a]	Case	Control	*p* - value[b]
$CD8^+$ T cells	0.05 ± 0.03	0.05 ± 0.03	0.345
$CD4^+$ T cells	0.14 ± 0.05	0.17 ± 0.06	**0.005**
NK cells	0.05 ± 0.03	0.06 ± 0.04	0.247
B cells	0.04 ± 0.02	0.04 ± 0.02	0.133
Monocytes	0.07 ± 0.02	0.07 ± 0.02	0.731
Granulocytes	0.66 ± 0.08	0.61 ± 0.07	**0.0001**

[a] Six different cell type proportions were estimated using Renius reference dataset available in the FlowStorted.Blood.450K Bioconductor package (v1.2.0)
[b]*p* values are calculated by t test, significant *p* values are in bold

Supplementary Table S2: Methylation difference of seven CpGs after cell proportion adjustment

Name	Gene	Mean β (control)	Mean β (case)	Δβ	*p*- value*
cg06418238	*RPTOR*	0.378	0.286	-0.092	**4.57E-06**
cg00736299	*MGRN1*	0.513	0.458	-0.055	**4.13E-07**
cg27466532	*RAPSN*	0.630	0.581	-0.049	**0.0019**
cg06526620	*FUT4*	0.460	0.414	-0.046	**0.0016**
cg21932542	*RADIL*	0.458	0.401	-0.057	**0.0005**
cg22941668	*MIR145*	0.491	0.440	-0.052	**0.0014**
cg22233512	*MSI2*	0.390	0.332	-0.058	**0.0163**

* *p*-value is the p-value for group calculated by beta regression, adjusted by age, batch and cell counts. Significant *p* values are in bold.

Supplementary Table S3: Association between methylation levels in *RPTOR* (cg06418238), *MGRN1* (cg00736299), *RAPSN* (cg27466532) and breast cancer risk in three validation cohorts

Genes	Quartiles (methylation range)	Control N	Case N	OR (95% CI)	*p* value[a]
Validation I					
RPTOR (cg06418238)	Q4(≥0.31)	37	21	1.00	
	Q3(0.23-0.31)	26	20	1.36 (0.61-2.99)	0.452
	Q2(0.17-0.23)	25	26	1.83 (0.85-3.95)	0.122
	Q1(≤0.17)	14	42	5.29 (2.36-11.86)	**5.35E-05**
MGRN1 (cg00736299)	Q4(≥0.47)	37	17	1.00	
	Q3(0.34-0.47)	26	25	2.09 (0.94-4.63)	0.068
	Q2(0.24-0.34)	25	27	2.35 (1.07-5.19)	**0.034**
	Q1(≤0.24)	14	40	6.22 (2.69-14.36)	**1.86E-05**
RAPSN (cg27466532)	Q4(≥0.66)	30	24	1.00	
	Q3(0.56-0.66)	34	15	0.55 (0.25-1.24)	0.150
	Q2(0.45-0.56)	22	32	1.82 (0.85-3.90)	0.125
	Q1(≤0.45)	16	38	2.97 (1.34-6.56)	**0.007**
Validation II					
RPTOR (cg06418238)	Q4(≥0.39)	65	32	1.00	
	Q3(0.22-0.39)	39	50	2.60 (1.44-4.72)	**0.002**
	Q2(0.11-0.22)	35	59	3.42 (1.89-6.21)	**5.02E-05**
	Q1(≤0.11)	50	48	1.95 (1.09-3.48)	**0.024**
MGRN1 (cg00736299)	Q4(≥0.68)	70	24	1.00	
	Q3(0.36-0.68)	43	47	3.19 (1.71-5.93)	**2.55E-04**
	Q2(0.09-0.36)	32	67	6.11 (3.26-11.42)	**1.50E-08**
	Q1(≤0.09)	44	50	3.31 (1.79-6.14)	**1.36E-04**
RAPSN (cg27466532)	Q4(≥0.86)	56	39	1.00	
	Q3(0.59-0.86)	41	51	1.79 (1.00-3.19)	**0.050**
	Q2(0.29-0.59)	39	58	2.14 (1.20-3.80)	**0.010**
	Q1(≤0.29)	53	41	1.11 (0.62-1.98)	0.721
Validation III					
RPTOR (cg06418238)	Q4(≥0.38)	79	54	1.00	
	Q3(0.29-0.38)	57	62	1.59 (0.97-2.62)	0.068
	Q2(0.21-0.29)	64	68	1.55 (0.96-2.53)	0.075
	Q1(≤0.21)	50	86	2.52 (1.54-4.11)	**2.31E-04**
MGRN1 (cg00736299)	Q4(≥0.59)	89	40	1.00	
	Q3(0.41-0.59)	68	57	1.87 (1.12-3.12)	**0.017**
	Q2(0.26-0.41)	49	82	3.72 (2.23-6.23)	**5.42E-07**
	Q1(≤0.26)	43	89	4.61 (2.73-7.76)	**9.33E-09**
RAPSN (cg27466532)	Q4(≥0.70)	78	59	1.00	
	Q3(0.60-0.70)	60	52	1.15 (0.69-1.89)	0.595
	Q2(0.49-0.60)	61	80	1.72 (1.08-2.79)	**0.023**
	Q1(≤0.49)	51	79	2.05 (1.26-3.34)	**0.004**

[a] *p* values are calculated by logistic regression and adjusted for age, significant *p* values are in bold

Supplementary Table S4: Correlation of cg06418238, cg00736299 and cg27466532 methylation with clinical features in sporadic BC patients

Characteristics	Group	N	Median methylation levels		
			RPTOR (cg06418238)	*MGRN1* (cg00736299)	*RAPSN* (cg27466532)
Age[a]	Q1 (< 45.47)	85	0.19	0.29	0.53
	Q2 (45.47-50.86)	88	0.20	0.29	0.53
	Q3 (> 50.86 - 61.52)	87	0.18	0.28	0.54
	Q4 (> 61.52)	86	0.24	0.31	0.52
	p value		0.259	0.668	0.974
Menopause status[a]	premenopausal	132	0.19	0.28	0.53
	perimenopausal	32	0.20	0.25	0.53
	postmenopausal	167	0.21	0.31	0.53
	p value		0.239	0.249	0.636
ER status[b c]	negative	47	0.23	0.27	0.49
	postive	289	0.19	0.29	0.54
	p value		0.102	0.434	0.287
PR status[b c]	negative	73	0.19	0.29	0.52
	postive	264	0.20	0.29	0.53
	p value		0.832	0.944	0.712
HER2 status[b d]	negative	282	0.20	0.28	0.53
	postive	54	0.22	0.31	0.53
	p value		0.545	0.579	0.605
Histological tumor grading[a]	I	55	0.18	0.30	0.52
	II	208	0.20	0.29	0.54
	III	74	0.19	0.28	0.52
	p value		0.476	0.905	0.725
Tumor size[a]	IS(*in situ*) and pT1	198	0.20	0.27	0.53
	pT2	116	0.20	0.29	0.54
	pT3 and pT4	24	0.16	0.36	0.51
	p value		0.326	0.261	0.620
lymph node status[b]	N0	236	0.20	0.28	0.525
	N1-N3	97	0.19	0.32	0.53
	p value		0.191	0.268	0.895
Stage[a]	0 and I	163	0.21	0.26	0.52
	II	131	0.19	0.29	0.56
	III and IV	45	0.21	0.34	0.52
	p value		0.202	0.169	0.645

[a b] Tests performed are[a] Jonckheere-Terpstra test and [b] Wilcoxon rank sum test, depending on the type of variable
[c] Immunoreactive score (IRS) 0–2 was defined as ER/PR negative and 3–12 as ER/PR positive
[d] HER-2 IHC-score 0–1 was defined as HER2 negative and 3 as definitely positive. An IHC-score equal to 2 was further analyzed by FISH/CISH and deemed positive if HER2 was amplified

Supplementary Table S5: Methylation differences of CpG sites in *RPTOR*, *MGRN1* and *RAPSN* in different types of leucocytes

See Supplementary File 1

Supplementary Table S6: Sequences of the target amplicons

Amplicon	Sequence[a]
RPTOR	GTGGGGCCTCTGCAGCAGCTGAGACGTTTGGCAGTTTCTTCAAGACTTGGAG<br>TAAAACCT***CG***CCTGAGATAGAATCTAAATACACTGGGCTTGGATCGCCCATTTGA<br>CTAAATTTCGATCCCAACTCTAGCTTTCAGAAACCTCCTTATAGGGAGAGACCTA<br>TTGCTATGGGAAGGCAAGAGAAATTAATGTCTGCGTTATTCCAGCAAAGCCCCTG<br>CACTGCGCTGGAGGGACTTTTGTTTGCGTCTTTGGGTTGCAGAAGGGGCTATGAT<br>TGTGCGTGGCTTATCTTGGTCTCCAGCCCAGTTTAGACCTCCTTCTAG<br>CTGATCTCTTCTCTCACTTGTACTTTGGAAGAGAGTCAGGGC<br>TTGGCTTTGGGTCACCA
MGRN1	CCCTGGGGCACAAGGGAAGCCCAAGGAAACCAAAAAGCTCAGCCCAA<br>GGAGCTCGAACCCGCAGGGTAAAGGAG***CG***GGACACGGGGCCAAGAAGCAT<br>GCGCGGGGCGCGATTGCAAAACGCAGCAGCGACGTTAGTTCCGCCT<br>GCAACGTCCATTCCACTGCCAGCAAGAAAAAAAAGGGAAACGGCACGGGG<br>GCCACGCGGGGCGGAGGCAAAGCTGAGGGGAGGGCGGCGGGGCCAGC<br>GCACCTGGCGTTTCGCCCACGGACCGAGAGCTTTTCCGGTTCCACAAACTCC<br>CTGGGCAGGGAGGCCGGGACTGGGCAAGTGGAGCTGACGGGTTATATGA<br>ATCAGACACTAGCCTGGGGCGGAAACGATCTCCCCTACTTGTCGTGGGTT<br>TAAAAATGAGGGCGGGGGCAAAAAAACGTAGCCGGAGGACCAGACACTTA<br>TGTTTCCCTTTTAGGTTTCTTGCTGGCCAGG
RAPSN	GACCTCCAGCTGGTGAGAGGCCTGAGCTCTAGCCTGGCTTCCTTCGA<br>ACTCTCAGTGGGACCCTAGCCAAGTGACCTGGCACCGTGGAC<br>TGAAGAGAGGAATGGTACCTGGCTCACTGGACTGCTATGAGAACTCAATGA<br>GAAAGAGTTGAGAAGGACTTAGGACAGTGCCCGGCACAGGAAACCCCG<br>**C**ATGTGCTGCAAGAGGCTATTTTGCTCCATGGCAAATCCCCACC***C***<br>***G***GGCCAGAACCCAGCCCTTGAAGTTTCAATGTCTCACTTCCAATCCCAACCCCG<br>GGCTGAAAGGGTCCTCATAATGAGATTTAACACCAGATACTGTGGAGAAGGG<br>CAAGGTCTTATGTAGTATTGGGGCCCCCGCTAGCAGGCCAGCTCATCCAGCCCCC<br>CTTTCTCCTCTCTCTGCATGCCAGCCTTCTTCCTGCCCCAGG<br>GCAAAAGTTTATCCTTTCTGGCTGGGCAATTCAGTGGTTTT

[a] The CpG sites which were discovered by 450K array are underlined and marked in italic and bold. All other CpG sites that could be measured are underlined
