# Supplementary material for: DNA methylation array analysis identifies breast cancer associated RPTOR, MGRN1 and RAPSN hypomethylation in peripheral blood DNA
Source: Oncotarget. 2016 Aug 26;7(39):64191–202. doi: 10.18632/oncotarget.11640 (PMC5325435; doi:10.18632/oncotarget.11640)
Supplement: Supplementary file 2 [file oncotarget-07-64191-s002.doc]

| **Table S5:** Methylation differences of CpG sites in *RPTOR*, *MGRN1* and *RAPSN* in different types of leucocytes | | | | |
| --- | --- | --- | --- | --- |
| CpG sites | Cell population | Control Median (IQR) n=13 | Case Median (IQR) n=7 | *p* value^a^ |
| *RPTOR* |  |  |  |  |
|  | whole blood | 0.10 (0.06-0.14) | 0.09 (0.08-0.09) | 0.922 |
| RPTOR_CpG_1 | B cells | 0.55 (0.47-0.58) | 0.51 (0.46-0.62) | 0.856 |
|  | T cells | 0.30 (0.19-0.35) | 0.28 (0.19-0.35) | 0.799 |
|  | B/T-cells depleted leukocytes | 0.03 (0.03-0.06) | 0.03 (0.02-0.06) | 0.579 |
|  | whole blood | 0.28 (0.23-0.33) | 0.21 (0.18-0.26) | **0.027** |
| cg06418238 | B cells | 0.71 (0.64-0.73) | 0.71 (0.65-0.79) | 0.877 |
|  | T cells | 0.55 (0.40-0.60) | 0.50 (0.49-0.60) | 0.799 |
|  | B/T-cells depleted leukocytes | 0.11 (0.08-0.21) | 0.12 (0.10-0.14) | 0.579 |
|  | whole blood | 0.66 (0.60-0.67) | 0.64 (0.58-0.67) | 0.278 |
| RPTOR_CpG_3 | B cells | 0.95 (0.90-0.99) | 0.92 (0.86-0.93) | 0.255 |
|  | T cells | 0.89 (0.75-0.92) | 0.84 (0.77-0.88) | 0.376 |
|  | B/T-cells depleted leukocytes | 0.53 (0.50-0.60) | 0.53 (0.51-0.58) | 0.769 |
|  | whole blood | 0.87 (0.81-0.90) | 0.93 (0.91-0.93) | 0.198 |
| RPTOR_CpG_4 | B cells | 1.00 (0.98-1.00) | 1.00 (0.97-1.00) | 0.856 |
|  | T cells | 1.00 (0.98-1.00) | 0.98 (0.95-1.00) | 0.376 |
|  | B/T-cells depleted leukocytes | 0.91 (0.90-0.92) | 0.81 (0.78-0.90) | 0.135 |
|  | whole blood | 0.81 (0.72-0.84) | 0.79 (0.76-0.82) | 1.000 |
| RPTOR_CpG_5 | B cells | 0.96 (0.90-1.00) | 0.98 (0.95-0.99) | 0.585 |
|  | T cells | 0.91 (0.88-0.98) | 0.88 (0.83-0.94) | 0.308 |
|  | B/T-cells depleted leukocytes | 0.71 (0.67-0.77) | 0.70 (0.68-0.75) | 0.492 |
|  | whole blood | 0.74 (0.71-0.79) | 0.67 (0.66-0.75) | 0.154 |
| RPTOR_CpG_8 | B cells | 0.91 (0.89-0.94) | 0.95 (0.91-0.95) | 0.400 |
|  | T cells | 0.88 (0.80-0.90) | 0.85 (0.83-0.86) | 0.224 |
|  | B/T-cells depleted leukocytes | 0.63 (0.61-0.67) | 0.64 (0.56-0.65) | 0.376 |
| *RAPSN* |  |  |  |  |
|  | whole blood | 0.95 (0.93-0.96) | 0.91 (0.87-0.94) | 0.091 |
| RAPSN_CpG_1 | B cells | 0.95 (0.94-0.98) | 0.97 (0.91-0.99) | 1.000 |
|  | T cells | 0.94 (0.93-0.96) | 0.96 (0.95-0.98) | 0.055 |
|  | B/T-cells depleted leukocytes | 0.94 (0.93-0.95) | 0.95 (0.93-0.95) | 0.858 |
|  | whole blood | 0.63 (0.53-0.74) | 0.45 (0.44-0.62) | 0.056 |
| RAPSN_CpG_2 | B cells | 0.81 (0.76-0.85) | 0.80 (0.78-0.82) | 0.592 |
|  | T cells | 0.76 (0.72-0.79) | 0.63 (0.54-0.81) | 0.198 |
|  | B/T-cells depleted leukocytes | 0.60 (0.50-0.63) | 0.56 (0.52-0.61) | 0.494 |
|  | whole blood | 0.41 (0.37-0.52) | 0.33 (0.30-0.41) | 0.103 |
| RAPSN_CpG_4 | B cells | 0.78 (0.73-0.89) | 0.81 (0.76-0.82) | 0.650 |
|  | T cells | 0.71 (0.45-0.86) | 0.68 (0.10-0.72) | 0.535 |
|  | B/T-cells depleted leukocytes | 0.28 (0.16-0.41) | 0.30 (0.28-0.32) | 0.802 |
|  | whole blood | 0.80 (0.74-0.84) | 0.54 (0.50-0.70) | **0.022** |
| RAPSN_CpG_5 | B cells | 0.89 (0.83-0.94) | 0.88 (0.87-0.94) | 0.967 |
|  | T cells | 0.86 (0.78-0.88) | 0.81 (0.73-0.86) | 0.376 |
|  | B/T-cells depleted leukocytes | 0.68 (0.65-0.74) | 0.68 (0.65-0.81) | 0.693 |
|  | whole blood | 0.56 (0.48-0.62) | 0.39 (0.38-0.53) | **0.047** |
| cg27466532 | B cells | 0.88 (0.84-0.94) | 0.91 (0.88-0.96) | 0.482 |
|  | T cells | 0.82 (0.68-0.86) | 0.62 (0.60-0.83) | 0.076 |
|  | B/T-cells depleted leukocytes | 0.49 (0.40-0.54) | 0.44 (0.37-0.46) | 0.134 |
|  | whole blood | 0.73 (0.67-0.79) | 0.65 (0.54-0.69) | 0.055 |
| RAPSN_CpG_7 | B cells | 0.77 (0.73-0.85) | 0.76 (0.74-0.84) | 0.592 |
|  | T cells | 0.75 (0.71-0.83) | 0.75 (0.72-0.80) | 0.922 |
|  | B/T-cells depleted leukocytes | 0.70 (0.62-0.75) | 0.66 (0.64-0.71) | 0.494 |
| RAPSN_CpG_8 | whole blood | 0.96 (0.93-0.98) | 0.96 (0.93-0.97) | 0.630 |
|  | B cells | 0.97 (0.96-0.98) | 0.97 (0.97-0.98) | 0.482 |
|  | T cells | 0.94 (0.93-0.97) | 0.94 (0.93-0.97) | 0.922 |
|  | B/T-cells depleted leukocytes | 0.95 (0.93-0.96) | 0.94 (0.93-0.95) | 0.590 |

| **Table S5** continued | | | | |
| --- | --- | --- | --- | --- |
| CpG sites | Cell population | Control Median (IQR) n=13 | Case Median (IQR) n=7 | *p* value^a^ |
| *MGRN1* |  |  |  |  |
|  | whole blood | 0.23 (0.15-0.47) | 0.21 (0.11-0.28) | 0.285 |
| MGRN1_CpG_1 | B cells | 0.49 (0.37-0.63) | 0.53 (0.34-0.68) | 0.896 |
|  | T cells | 0.53 (0.36-0.60) | 0.50 (0.21-0.91) | 0.741 |
|  | B/T-cells depleted leukocytes | 0.07 (0.02-0.10) | 0.12 (0.02-0.18) | 0.750 |
|  | whole blood | 0.76 (0.71-0.77) | 0.65 (0.62-0.74) | 0.475 |
| MGRN1_CpG_2 | B cells | 0.95 (0.87-1.00) | 0.89 (0.86-0.91) | 0.197 |
|  | T cells | 0.89 (0.78-0.91) | 0.85 (0.80-0.94) | 0.801 |
|  | B/T-cells depleted leukocytes | 0.61 (0.58-0.63) | 0.59 (0.49-0.61) | 0.146 |
|  | whole blood | 0.58 (0.43-0.64) | 0.29 (0.26-0.32) | 0.008 |
| cg00736299 | B cells | 0.87 (0.79-0.90) | 0.83 (0.78-0.86) | 0.236 |
|  | T cells | 0.79 (0.66-0.87) | 0.72 (0.67-0.79) | 0.572 |
|  | B/T-cells depleted leukocytes | 0.24 (0.18-0.26) | 0.23 (0.14-0.38) | 0.710 |
|  | whole blood | 0.47 (0.34-0.55) | 0.26 (0.24-0.28) | 0.142 |
| MGRN1_CpG_4 | B cells | 0.79 (0.77-0.85) | 0.88 (0.79-0.91) | 0.349 |
|  | T cells | 0.85 (0.71-0.90) | 0.73 (0.53-0.81) | 0.709 |
|  | B/T-cells depleted leukocytes | 0.23 (0.09-0.24) | 0.16 (0.03-0.24) | 0.313 |
|  | whole blood | 0.50 (0.33-0.53) | 0.27 (0.25-0.35) | 0.132 |
| MGRN1_CpG_5.6.7.8 | B cells | 0.75 (0.71-0.79) | 0.75 (0.69-0.76) | 0.327 |
|  | T cells | 0.66 (0.58-0.74) | 0.64 (0.61-0.70) | 0.590 |
|  | B/T-cells depleted leukocytes | 0.21 (0.15-0.27) | 0.20 (0.12-0.33) | 0.319 |
|  | whole blood | 0.31 (0.26-0.41) | 0.13 (0.10-0.19) | 0.383 |
| MGRN1_CpG_11 | B cells | 0.53 (0.38-0.59) | 0.59 (0.45-0.75) | 0.200 |
|  | T cells | 0.37 (0.25-0.41) | 0.45 (0.37-0.45) | 0.302 |
|  | B/T-cells depleted leukocytes | 0.09 (0.05-0.09) | 0.15 (0.05-0.30) | 1000 |
|  | whole blood | 0.50 (0.39-0.59) | 0.35 (0.27-0.41) | 0.234 |
| MGRN1_CpG_12 | B cells | 0.95 (0.91-0.98) | 0.90 (0.85-0.95) | 0.508 |
|  | T cells | 0.72 (0.67-0.92) | 0.76 (0.65-0.83) | 0.804 |
|  | B/T-cells depleted leukocytes | 0.27 (0.13-0.37) | 0.19 (0.09-0.38) | 0.845 |
|  | whole blood | 0.76 (0.71-0.77) | 0.65 (0.62-0.74) | 0.475 |
| MGRN1_CpG_13 | B cells | 0.95 (0.87-1.00) | 0.89 (0.86-0.91) | 0.197 |
|  | T cells | 0.89 (0.78-0.91) | 0.85 (0.80-0.94) | 0.801 |
|  | B/T-cells depleted leukocytes | 0.61 (0.58-0.63) | 0.59 (0.49-0.61) | 0.146 |
|  | whole blood | 0.44 (0.36-0.53) | 0.31 (0.28-0.31) | 0.456 |
| MGRN1_CpG_14 | B cells | 0.65 (0.60-0.72) | 0.67 (0.62-0.75) | 0.414 |
|  | T cells | 0.52 (0.46-0.56) | 0.51 (0.44-0.64) | 0.563 |
|  | B/T-cells depleted leukocytes | 0.25 (0.18-0.31) | 0.19 (0.06-0.38) | 0.892 |
|  | whole blood | 0.52 (0.40-0.66) | 0.33 (0.31-0.34) | 0.081 |
| MGRN1_CpG_15 | B cells | 0.91 (0.80-0.94) | 0.83 (0.81-0.87) | 0.815 |
|  | T cells | 0.73 (0.62-0.83) | 0.76 (0.67-0.84) | 0.341 |
|  | B/T-cells depleted leukocytes | 0.26 (0.12-0.30) | 0.26 (0.24-0.32) | 0.618 |
|  | whole blood | 0.49 (0.39-0.61) | 0.29 (0.27-0.33) | 0.080 |
| MGRN1_CpG_16.17.18 | B cells | 0.84 (0.81-0.88) | 0.80 (0.78-0.88) | 0.785 |
|  | T cells | 0.85 (0.60-0.89) | 0.74 (0.65-0.78) | 0.321 |
|  | B/T-cells depleted leukocytes | 0.23 (0.16-0.28) | 0.22 (0.15-0.36) | 0.717 |
|  | whole blood | 0.58 (0.44-0.64) | 0.39 (0.35-0.47) | 0.122 |
| MGRN1_CpG_19.20 | B cells | 0.91 (0.84-0.94) | 0.89 (0.83-0.93) | 0.755 |
|  | T cells | 0.69 (0.67-0.88) | 0.77 (0.66-0.82) | 0.617 |
|  | B/T-cells depleted leukocytes | 0.35 (0.27-0.37) | 0.28 (0.17-0.39) | 0.160 |
|  | whole blood | 0.44 (0.36-0.53) | 0.31 (0.28-0.31) | 0.456 |
| MGRN1_CpG_21 | B cells | 0.65 (0.60-0.72) | 0.67 (0.62-0.75) | 0.414 |
|  | T cells | 0.52 (0.46-0.56) | 0.51 (0.44-0.64) | 0.563 |
|  | B/T-cells depleted leukocytes | 0.25 (0.18-0.31) | 0.19 (0.06-0.38) | 0.892 |
|  | whole blood | 0.50 (0.38-0.61) | 0.26 (0.23-0.29) | **0.026** |
| MGRN1_CpG_22.23 | B cells | 0.80 (0.69-0.83) | 0.78 (0.63-0.81) | 0.726 |
|  | T cells | 0.60 (0.58-0.79) | 0.67 (0.61-0.73) | 0.362 |
|  | B/T-cells depleted leukocytes | 0.27 (0.22-0.31) | 0.26 (0.17-0.34) | 0.618 |
|  | whole blood | 0.49 (0.35-0.64) | 0.31 (0.29-0.39) | 0.074 |
| MGRN1_CpG_26 | B cells | 0.86 (0.84-0.89) | 0.79 (0.72-0.87) | 0.293 |
|  | T cells | 0.70 (0.61-0.79) | 0.67 (0.58-0.72) | 0.457 |
|  | B/T-cells depleted leukocytes | 0.24 (0.17-0.32) | 0.23 (0.13-0.39) | 0.341 |
|  | whole blood | 0.60 (0.44-0.70) | 0.31 (0.29-0.39) | **0.026** |
| MGRN1_CpG_27 | B cells | 0.91 (0.87-0.96) | 0.90 (0.87-0.96) | 0.459 |
|  | T cells | 0.73 (0.61-0.85) | 0.75 (0.63-0.75) | 0.934 |
|  | B/T-cells depleted leukocytes | 0.22 (0.15-0.27) | 0.24 (0.12-0.36) | 0.365 |
|  | whole blood | 0.46 (0.42-0.60) | 0.33 (0.29-0.37) | 0.236 |
| MGRN1_CpG_28 | B cells | 0.78 (0.74-0.80) | 0.77 (0.74-0.84) | 0.370 |
|  | T cells | 0.62 (0.54-0.71) | 0.64 (0.56-0.77) | 0.869 |
|  | B/T-cells depleted leukocytes | 0.24 (0.14-0.28) | 0.23 (0.14-0.35) | 0.389 |
|  | whole blood | 0.62 (0.48-0.66) | 0.40 (0.36-0.49) | 0.132 |
| MGRN1_CpG_29 | B cells | 0.96 (0.90-1.00) | 0.92 (0.87-0.99) | 0.696 |
|  | T cells | 0.75 (0.73-0.93) | 0.77 (0.72-0.83) | 0.555 |
|  | B/T-cells depleted leukocytes | 0.27 (0.22-0.35) | 0.33 (0.21-0.44) | 0.440 |
|  | whole blood | 0.50 (0.42-0.63) | 0.37 (0.33-0.46) | 0.132 |
| MGRN1_CpG_31 | B cells | 0.86 (0.82-0.88) | 0.83 (0.78-0.91) | 0.755 |
|  | T cells | 0.63 (0.55-0.75) | 0.68 (0.58-0.69) | 0.619 |
|  | B/T-cells depleted leukocytes | 0.26 (0.21-0.30) | 0.29 (0.15-0.40) | 0.414 |
|  | whole blood | 0.37 (0.26-0.58) | 0.31 (0.24-0.38) | 0.781 |
| MGRN1_CpG_32 | B cells | 0.88 (0.77-0.94) | 0.83 (0.78-0.90) | 0.815 |
|  | T cells | 0.55 (0.45-0.83) | 0.66 (0.39-0.76) | 0.837 |
|  | B/T-cells depleted leukocytes | 0.18 (0.14-0.27) | 0.22 (0.10-0.38) | 0.786 |
|  | whole blood | 0.52 (0.50-0.55) | 0.42 (0.38-0.54) | 0.641 |
| MGRN1_CpG_34 | B cells | 0.83 (0.77-0.98) | 0.84 (0.76-0.94) | 0.969 |
|  | T cells | 0.70 (0.69-0.76) | 0.72 (0.55-0.73) | 0.967 |
|  | B/T-cells depleted leukocytes | 0.24 (0.20-0.32) | 0.23 (0.10-0.33) | 0.365 |

^a^ *p* values are calculated by Mann-Whitney Test, significant *p* values are in bold
